# Protection afforded by previous *Vibrio cholerae* infection against subsequent disease and infection: A review

**DOI:** 10.1371/journal.pntd.0009383

**Published:** 2021-05-20

**Authors:** Tiffany Leung, Laura Matrajt

**Affiliations:** Vaccine and Infectious Disease Division, Fred Hutchinson Cancer Research Center, Seattle, Washington, United States of America; Washington University School of Medicine, UNITED STATES

## Abstract

**Background:**

Cholera is an acute, diarrheal disease caused by *Vibrio cholerae* O1 or 139 that is associated with a high global burden.

**Methods:**

We analyzed the estimated duration of immunity following cholera infection from available published studies. We searched PubMed and Web of Science for studies of the long-term immunity following cholera infection. We identified 22 eligible studies and categorized them as either observational, challenge, or serological.

**Results:**

We found strong evidence of protection at 3 years after infection in observational and challenge studies. However, serological studies show that elevated humoral markers of potential correlates of protection returned to baseline within 1 year. Additionally, a subclinical cholera infection may confer lower protection than a clinical one, as suggested by 3 studies that found that, albeit with small sample sizes, most participants with a subclinical infection from an initial challenge with cholera had a symptomatic infection when rechallenged with a homologous biotype.

**Conclusions:**

This review underscores the need to elucidate potential differences in the protection provided by clinical and subclinical cholera infections. Further, more studies are warranted to bridge the gap between the correlates of protection and cholera immunity. Understanding the duration of natural immunity to cholera can help guide control strategies and policy.

## Introduction

Cholera is an acute, diarrheal disease caused by the gram-negative bacteria *Vibrio cholerae*, which encompasses over 200 serogroups [1], but only serogroups O1 and O139 are known to cause cholera in humans. The O1 serogroup can be classified into 2 biotypes, classical and El Tor, and into 2 major serotypes, Ogawa and Inaba [2]. There have been 7 recorded cholera pandemics; the first 6 all caused by the classical biotype, and the current one, ongoing since 1961, caused by El Tor biotype [3]. Both classical and El Tor biotypes coexisted between the 1970s and 1980s, and since El Tor had completely replaced the classical biotype [4]. The reasons for the disappearance of the classical biotype remain unknown.

The clinical spectrum of *V*. *cholerae* infections ranges from asymptomatic colonization to severe diarrhea leading to life-threatening dehydration [2,3]. Cholera remains endemic in at least 47 countries [1], resulting in an estimated 1.3 to 4.0 million cases and 21,000 to 143,000 deaths annually worldwide [5]. Large outbreaks of cholera have been documented following natural disasters, in war zones [6,7], and more recently in sub-Saharan Africa [8]. Current interventions to control and prevent cholera include vaccines, oral rehydration solutions, improvement to health systems, and investments in water, sanitation, and hygiene [9]. In 2017, the Global Task Force on Cholera Control set a goal to reduce cholera deaths by 90% and eliminate disease transmission in up to 20 countries by 2030 [9].

Understanding the natural history of cholera infection, particularly the mechanisms responsible for long-term protection, is key to prevention, outbreak response, and cholera control. However, no clear serological correlate of protection has been identified for cholera and different methodologies provide different estimates of duration of protection.

Observational studies of cholera analyze population-level surveillance data [10–13]. Comparatively, human challenge studies of cholera involve purposefully exposing healthy participants to *V*. *cholerae* and taking fecal and serologic samples to confirm an infection and measure diverse immune responses [14,15]. These challenge studies contribute insight into the duration of protection against infection and disease, existence of subclinical infections, and potential presence of cross-immunity between serotypes [16–18]. Most of them were done in the 1980s when the classical *V*. *cholerae* was the circulating biotype, and most used classical biotypes for the challenges. They generally comprise a small number of participants in the United States, where cholera is nonendemic, and months of follow-up [15–17].

Serological markers could provide a qualitative indication of protection against cholera. Indeed, following cholera infection, antibodies can be detected against several *V*. *cholerae* antigens, including the lipopolysaccharide (LPS) and B subunit of cholera toxin (CTB). Immunity against *V*. *cholerae* is serogroup-specific, as differentiated by the O-specific polysaccharide (OSP) component of LPS [19]. The best-characterized indirect marker of immunity to cholera is the serum vibriocidal antibody titer [20,21]. In household contacts of cholera patients, a higher baseline vibriocidal titer was associated with protection from *V*. *cholerae* O1 but not from *V*. *cholerae* O139 [20,22,23]. However, there is no threshold titer above which protection from *V*. *cholerae* has been established [13,22], and protection can persist even after vibriocidal levels return to baseline [20,24]. More recently, *V*. *cholerae*-specific memory B cells (MBCs), specifically LPS-specific IgG MBCs and OSP-specific MBCs, have been found in circulation after cholera infection and have been suggested as key players in maintaining long-term immunity [24–27].

Knowledge of the duration of protection is valuable to public health, epidemiological studies, and mathematical modeling studies. The estimates of protection following infection with *V*. *cholerae* obtained from observational, challenge, and serological studies form a biological basis used to inform mathematical models of cholera transmission. Mathematical models are increasingly used to inform policy, and wide model parameter ranges can result in wide-ranging and seemingly contradictory predictions. Currently, estimates of naturally acquired cholera immunity span wide, ranging from a few months to 9 years [28–30]. A better understanding of the duration of protection is then critical as we seek to optimize intervention strategies in the control of cholera outbreaks.

Here, we conduct a review of the published literature assessing the long-term immunity following cholera infection. We examine challenge, observational and postinfection serological studies to assess the overlap and differences in the estimated duration of protection following cholera infection.

## Methods

### Search strategy and selection criteria

We conducted this study following the PRISMA (Preferred Reporting Items for Systematic Reviews and Meta-Analyses) guidelines [31]. On January 17, 2020, we searched PubMed and Web of Science for articles in the English language published between January 1, 1960 and December 31, 2019. We searched PubMed with the string: “cholera”[title] OR “cholerae”[title] AND (natural OR immunity OR immune OR immunologic* OR immun*). Because immun* included many words unrelated to the purposes of this study, we specifically included the words immunity, immune, and immunologic. The search was then refined to human studies. In Web of Science, we used for articles with search string: Title = (cholera OR cholerae) AND Topic = (cholera) AND Topic = (“natural immunity” OR immunity OR immunologic OR immune). We then excluded results in categories “biochemistry molecular biology,” “genetics heredity,” or “veterinary sciences.”

After removing duplicates, one author (TL) screened all titles and abstracts to determine eligibility for full-text assessment. Mathematical modeling studies and studies without data on natural infection of *V*. *cholerae* were excluded from analysis. The titles for full-text assessment were reviewed by another author (LM). Reviewal of citations within each included study led to 11 additional studies included for full-text assessment. Any discrepancies on the study selection were resolved by discussion and consensus.

### Assessment

Full-text articles were classified as (i) an observational study; (ii) a challenge study, where participants received a subsequent challenge after an initial challenge with *V*. *cholerae*; (iii) a postinfection immune persistence study (henceforth referred to as persistence study), where potential immunological markers of long-term protection of cholera were measured and assessed; and (iv) other—meeting none of the above criteria and thus excluded from analysis. Because we were interested in long-term immunity to cholera, we excluded persistence and challenge studies with shorter than 3 months of follow-up or time interval between challenges. Challenge studies mostly used an infecting dose of 10^4^ to 10^6^ colony-forming units (CFU).

We focused on persistence studies that measured serum vibriocidal antibody titer and MBC responses specific to LPS and OSP of IgG isotype. We compared the protection from cholera observed in challenge and observational studies and contrasted that to the measurements and longevity of potential immunological markers in persistence studies. Because no long-term protection has been associated with CTB-specific IgA and IgG MBCs, LPS-specific IgA MBCs, or OSP-specific IgA and IgM MBCs [26,32], the analysis of these markers are found in Figs A-C in S1 Appendix.

## Results

**Fig 1:**
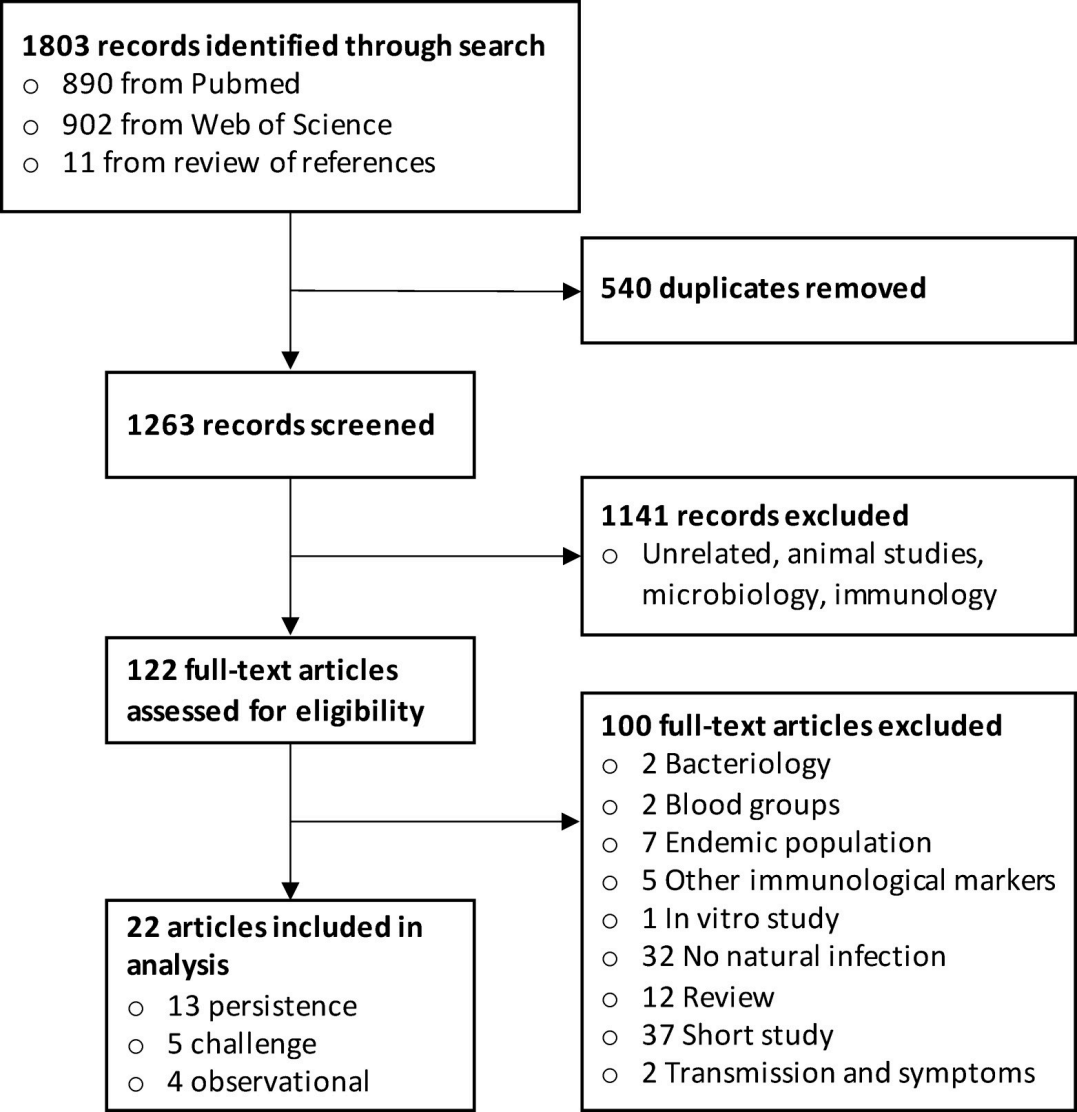
Study selection flow diagram for search process.

The literature search yielded 1,803 records (Fig 1). After excluding 1,777 records, 22 studies (4 observational, 5 challenge, and 13 persistence) met the inclusion criteria (Table 1). Here, we define a cholera infection as a positive stool culture for *V*. *cholerae* (irrespective of symptoms), a clinical cholera infection (clinical cholera) as a positive stool culture for *V*. *cholerae* accompanied by diarrhea, and a subclinical cholera infection as a positive stool culture without diarrhea.

**Table 1:** Details of studies that were included in this review.

Source (First author surname, year)	Location	Study type	Sample size	Age range	Study duration^b^	Measured response^a^
Aktar 2016 [27]	Bangladesh	Persistence	60	2–55	6	VC, OSP-IgG MBC, LPS-IgG MBC, Other
Alam 2011 [33]	Bangladesh	Persistence	37	≥18	12	VC, LPS-IgG MBC, Other
Alam 2013 [34]	Bangladesh	Persistence	30	18–46	12	LPS-IgG MBC, Other
Haney 2018 [21]	North America	Persistence	26	≥18	6	LPS-IgG MBC, Other
Harris 2009 [24]	Bangladesh	Persistence	29	5–59	12	VC, LPS-IgG MBC, Other
Hossain 2019 [35]	North America	Persistence	22	18–45	6	VC, LPS-IgG MBC, Other
Hossain 2019 [35]	Bangladesh	Persistence	13	18–45	6	VC, LPS-IgG MBC, Other
Jayasekera 2008 [36]	Bangladesh	Persistence	14	19–41^c^	3	VC, Other
Kendall 2010 [37]	Bangladesh	Persistence	32	1–44^c^	3	VC, LPS-IgG MBC, Other
McCormack 1969 [38]	Bangladesh	Persistence	130	not specified	6	Descriptive
Pierce 1970 [39]	not specified	Persistence	6	16–67	12–18	Descriptive
Snyder 1981 [40]	US	Persistence	7	not specified	9–11	Descriptive
Uddin 2011 [41]	Bangladesh	Persistence	7	22–44	12	VC, LPS-IgG MBC, Other
Uddin 2014 [42]	Bangladesh	Persistence	14	18–45	12	OSP-IgG MBC, LPS-IgG MBC, Other
Cash 1974 [14]	US	Challenge	21	≥18	4–12	
Hornick 1971 [18]	US	Challenge	13	≥18	3–12	
Levine 1980 [16]	US	Challenge	6	≥18	4–14 weeks	
Levine 1981 [17]	US	Challenge	4	≥18	33–36	
Morris 1995 [15]	US	Challenge	6	≥18	3	
Ali 2011 [10]	Bangladesh	Observational	NA	NA	NA	
Clemens 1991 [12]	Bangladesh	Observational	NA	NA	NA	
Glass 1982 [11]	Bangladesh	Observational	NA	NA	NA	
Woodward 1971 [43]	East Pakistan	Observational	NA	NA	NA	

^a^LSP- and OSP-specific MBCs of IgA and IgM; and CTB-specific MBCs of IgA, IgG, and IgM are denoted as “Other.”

^b^Months, unless specified.

^c^Listed as mean age ± SD.

IgG, immunoglobulin G; LPS, lipopolysaccharide; MBC, memory B cell; NA, not applicable; OSP, O-specific polysaccharide; VC, vibriocidal antibody.

### Observational studies

**Fig 2:**
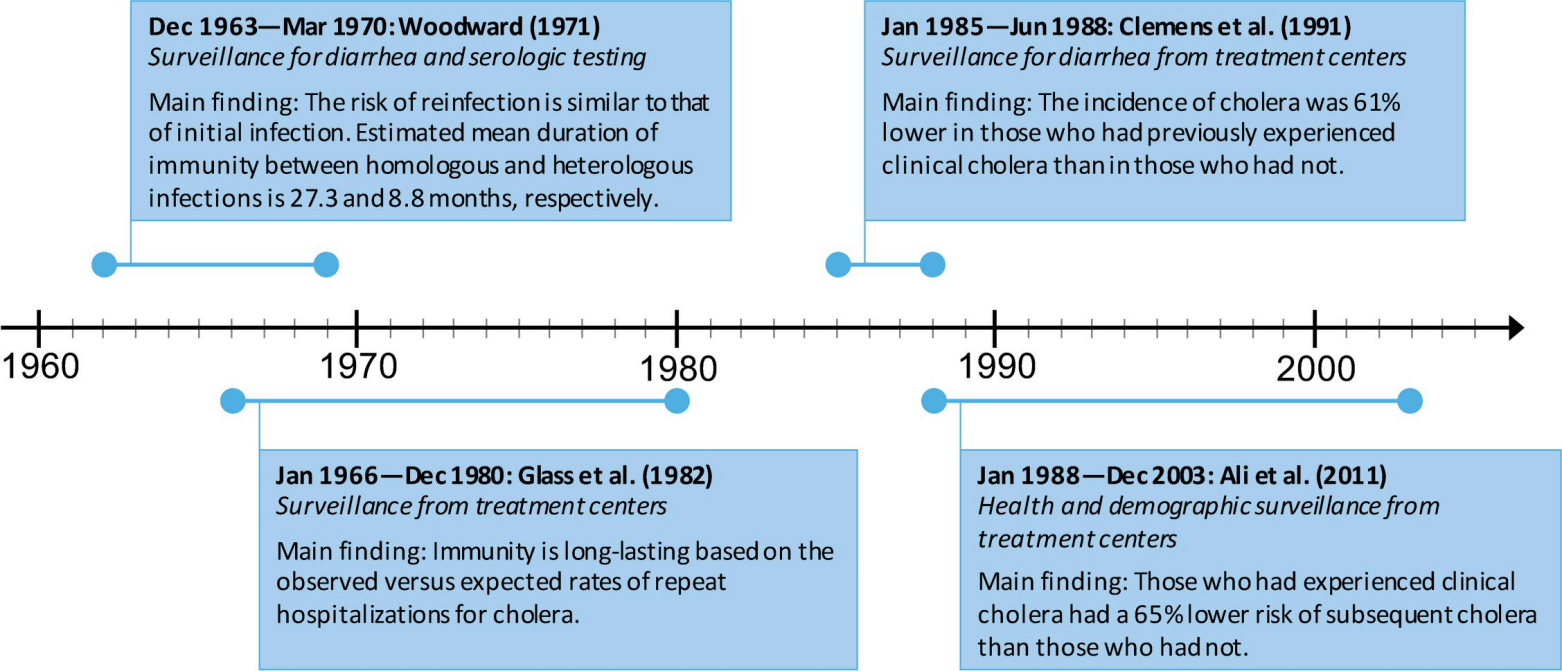
Main findings of the observational studies.

Four included observational studies examined surveillance data from 1963 to 2003 in East Pakistan (now Bangladesh) [43] and Bangladesh [10–12], where cholera remains endemic today (Fig 2). They corresponded hospitalizations and episodes of diarrhea to the risk of infection by *V*. *cholerae*.

#### Reduction in clinical cholera risk

Ali and colleagues [10] and Clemens and colleagues [12] found that, for patients in Bangladesh, clinical cholera was associated with a reduced risk of subsequent cholera. Clemens and colleagues found, from records collected over 42 months, the age-adjusted incidence of cholera was 61% (95% confidence interval [CI], 21% to 81%) lower in those who had had cholera than in those who had not [12]. Ali and colleagues reached similar conclusions but specific to the El Tor biotype. Those who had El Tor cholera had a 65% (95% CI, 37% to 81%) lower risk of subsequent El Tor cholera, irrespective of age, that was sustained over 3 years—the full study duration of the study from Ali and colleagues [10]. These findings were supported by Glass and colleagues [11], who found repeat hospitalizations were 90% lower than expected (only 3 repeats compared to 29 as expected from a life-table analysis).

Analyzed further by biotype, Clemens and colleagues [12] found that El Tor cholera was associated with “suggestive” protection with subsequent El Tor cholera (29% lower; 95% CI, −118% to 77%) but not with classical cholera (−6% lower; 95% CI, −182% to 60%). Classical cholera was associated with protection against clinical cholera after finding no cases of subsequent cholera following an initial clinical infection with classical *V*. *cholerae* [12]. Similarly, Ali and colleagues [10] did not detect any subsequent clinical cholera among those diagnosed with a classical *V*. *cholerae* infection, but in their study, classical cholera patients made up less than 1% of all cholera patients detected.

Unlike the 3 aforementioned studies that analyzed data collected at medical centers [10–12], Woodward analyzed 6 years of surveillance data that were maintained by daily house-to-house visits [43]. These data included subclinical and mild cholera infections, which hospital records would likely miss as patients were unlikely to seek treatment without severe clinical symptoms. Woodward [43] estimated similar rates of initial infection and reinfection (0.23% and 0.22%, respectively).

In records of cholera cases collected in Bangladesh between 1991 and 2000, Ali and colleagues [10] found, though not statistically significant, that initial O139 cholera was associated with a 63% (95% CI, −61% to 92%) lower risk of subsequent O139 cholera but no evidence of cross-protection between O1 and O139 serogroups.

#### Cross-protection across serotypes

Initial disease by El Tor *V*. *cholerae* was found 62% (95% CI, 11% to 84%) protective against subsequent El Tor cholera for at least 3 years [10]. Additionally, El Tor Inaba cholera was found to be protective against both El Tor Inaba and Ogawa cholera, whereas El Tor Ogawa cholera only protected against the homologous serotype.

Framing cross-protection as the mean time between infections, Woodward estimated the overall mean duration between cholera infections of Inaba and Ogawa serotypes was 19.3 months (range 1.5 to 60 months) [43]. The mean duration between serotype-homologous infections was longer than that between heterologous ones: 27.3 months (range 11 to 60 months) versus 8.8 months (range 1.5 to 29 months), respectively [43]. He estimated a lower annual reinfection rate for initial infections of Inaba (0.15%) than of Ogawa serotype (0.88%) [43]—in support that Inaba infections were associated with a lower risk of subsequent El Tor cholera than were Ogawa infections [10].

### Challenge studies

In the 5 included challenge studies (three of O1 classical [14,17,18], one of O1 El Tor [16], and one of O139 Bengal [15]), participants with clinical cholera (mild to severe diarrhea with a positive culture for *V*. *cholerae*) from their initial challenge were subsequently challenged with *V*. *cholerae* (Table 2).

**Table 2:** Details of challenge studies performed on individuals who experienced diarrhea on initial challenge.

Source	Sample size	Time to rechallenge^a^	Rechallenge biotype (and serotype)	Positive culture upon rechallenge (%)	Diarrhea upon rechallenge (%)	Dose in CFU (initial / rechallenge)
Cash et al. [14]	21	4–12	Classical (homologous)	1/21 (5)	0/21 (0)	10^6^ / 10^6^
Cash et al. [14]	6	4–12	Classical (heterologous)	5/6 (83)	4/6 (67)	10^6^ / 10^6^
Hornick et al. [18]	13	3–12	Classical (homologous)	0/13 (0)	0/13 (0)	10^4^–10^11^ / 10^4^–10^11^
Hornick et al. [18]	2	6–9	Classical (heterologous)	2/2 (100)	2/2 (100)	10^4^–10^11^ / 10^4^–10^11^
Levine et al. [16]	4	4–14 weeks	El Tor (homologous)	2/4 (50)	1/4 (25)	10^5^–10^6^ / 10^5^–10^6^
Levine et al. [16]	2	4–14 weeks	El Tor (heterologous)	0/2 (0)	0/2 (0)	10^5^ / 10^5^
Levine et al. [17]	4	33–36	Classical (3 heterologous)	1/4 (25)	0/4 (0)	10^6^ / 10^6^
Morris et al. [15]	6	3	O139 (homologous)	4/6 (67)	1/6 (17)	10^4^–10^6^ / 10^6^

The *V*. *cholerae* organisms used for rechallenge have matching biotype and either homologous or heterologous serotype to the ones used for initial challenge. Note that studies with more than 1 serotype are shown in separate lines.

^a^Months, unless specified.

#### O1 classical biotype

In a series of seminal studies by Levine and colleagues [16,17,44], 4 volunteers in the US were experimentally challenged with *V*. *cholerae* (classical) on 3 separate occasions: an initial challenge (presumably their first ever exposure to *V*. *cholerae*), followed by a second challenge 8 to 10 weeks later [16,44], and a third challenge 33 to 36 months after their initial challenge [17]. The 4 volunteers were completely protected for the second challenge, as confirmed by negative stool cultures for *V*. *cholerae* [16,17,44]. In the third challenge (3 heterologous serotype; 1 homologous serotype), none developed diarrhea, and one had a positive culture for *V*. *cholerae* [17]; it is unclear which participant had the positive culture. This study of 4 participants provides, at 3 years after the initial challenge (the longest follow-up to date for any published challenge study of cholera), evidence of protection against disease conferred by classical *V*. *cholerae*.

Two other challenge studies of classical cholera with rechallenges within 12 months reached similar conclusions (Table 2) [14,18]. When rechallenged with homologous serotype, all participants were protected from disease, with only one (1/21) in the Cash study (and none (0/13) in the Hornick study) with a positive stool culture for *V*. *cholerae* (Table 2). This contrasts with rechallenges of heterologous serotype, where most participants developed diarrhea and had a positive stool culture positive [14,18]. These findings suggest that classical cholera appears more effective at conferring protection against subsequent infection of homologous than of heterologous serotype. This is consistent with the findings of Woodward’s observational study [43], which estimated the average duration between homologous infections was longer than that between heterologous ones.

#### O1 El Tor biotype

We identified one challenge study of El Tor biotype in which 6 participants were initially challenged with serotypes Ogawa (*n =* 2) or Inaba (*n* = 4) and rechallenged with Inaba serotype 4 to 14 weeks later [16]. In the homologous rechallenge, one (1/4) participant developed diarrhea and another had positive stool cultures without clinical symptoms. In the heterologous rechallenge, both participants were free of infection, suggesting that El Tor Ogawa cholera may protect against El Tor Inaba cholera. Though the number of participants was very small, this challenge study suggests that El Tor cholera is effective at conferring some protection against heterologous and homologous rechallenges (for at least 4 to 14 weeks after initial cholera)—in agreement with the observational study of Ali and colleagues [10].

#### O139 Bengal biotype

One challenge study found that disease from *V*. *cholerae* O139 Bengal provided an 80% protective efficacy against subsequent disease from the same Bengal serotype but was not protective against reinfection as four (4/6) participants had positive stool cultures [15]. Ali and colleagues [10] reached a similar, although not statistically significant, conclusion that having O139 cholera was associated with a lower risk of subsequent O139 cholera over the first 3 years postinfection.

#### Subclinical infections

Subclinical infections, or infections confirmed by positive stool culture but unaccompanied by diarrhea, have been documented in these studies. This highlights the difference between protection from infection and protection from disease. The aforementioned challenges (Table 2) were done on participants with symptoms in their initial challenge.

We now summarize the findings of challenges performed on participants without diarrhea upon their initial challenge (Table 3). In 2 studies of classical *V*. *cholerae* by Cash and colleagues and Hornick and colleagues, 2 participants (2/3 and 2/2 each, respectively) experienced diarrhea after a serotype-homologous rechallenge under 12 months later [14,18]. Similar observations were reported on 3 participants without diarrhea on initial challenge with *V*. *cholerae* O139 Bengal [15]. When rechallenged with the same organisms 3 months later, one experienced diarrhea while the other two had subclinical infections and evidence of an antibody response [15]. Additionally, serum specimens from the volunteers challenged with classical *V*. *cholerae* showed that those with severe disease had demonstrable vibriocidal antibody titers, while those with subclinical infections failed to develop humoral antibodies [18].These studies, while having a very small number of participants, suggest that subclinical infections confer a less robust protection than clinical ones.

**Table 3:** Details of challenge studies performed on individuals who had not experienced diarrhea on initial challenge.

Source	Sample size	Time to rechallenge^a^	Rechallenge biotype (and serotype)	Positive culture upon rechallenge	Diarrhea upon rechallenge	Dose in CFU (initial / rechallenge)
Cash et al. [14]	3	4–12	Classical (homologous)	2/3	2/3	10^6^ / 10^6^
Hornick et al. [18]	2	6–9	Classical (heterologous)	2/2	2/2	10^4^–10^11^ / 10^4^–10^11^
Morris et al. [15]	3	3	O139 (homologous)	3/3	1/3	10^4^–10^6^ / 10^6^

The *V*. *cholerae* organisms used for rechallenge have matching biotype and either homologous or heterologous serotype to the ones used for initial challenge.

^a^Months.

### Persistence studies

We identified 13 persistence studies in which sera were collected and measured from residents in Bangladesh (10) [24,27,33–38,41,42], North America (3) [21,35,40], and an unspecified area (assumed India; 1) [39]. The study populations of several studies are notable. First, one study [35] compared the immune responses of 2 groups, cholera patients in Bangladesh and volunteers in North America. Second, one study included a group of North Americans who had naturally acquired cholera from contaminated food [40] (as opposed to being experimentally infected). Finally, in 2 studies comprising both vaccine and placebo groups, we report the results of the latter [21,42].

#### Vibriocidal responses

Ten of the 13 persistence studies measured the serum vibriocidal antibody response after infection with *V*. *cholerae*. We subdivided these studies into 2 groups by the main type of analysis performed within each study: statistical (7) and descriptive (3). The statistical group determined differences using statistical tests, while the descriptive group used titer fold differences.

**Fig 3:**
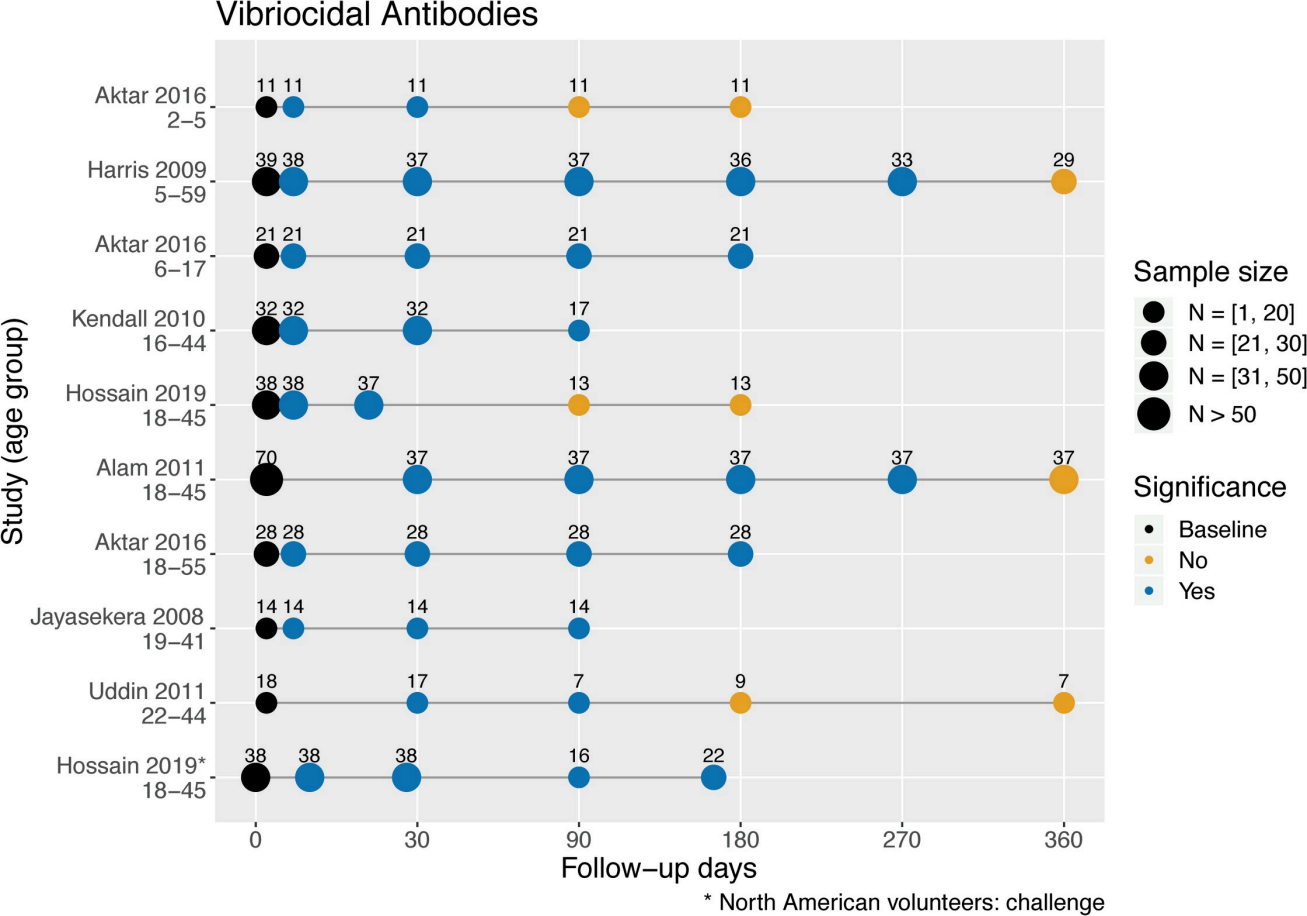
Serum vibriocidal antibody responses in cholera patients in Bangladesh by study and age range. Responses stratified by age are shown on different lines, and sample sizes may differ throughout various follow-up days. Within a study where the sample size of a follow-up day was unspecified, we set it equal to the smallest sample size of the follow-up days within the study (the most conservative estimate). Statistically significant differences in geometric mean titers of vibriocidal antibodies (*P* ≤ 0.05, two-tailed) were compared to baseline.

The first group of 7 studies compared samples at different time points to the baseline using statistical evidence of significant difference as reflected in the *P* value (Fig 3) [24,27,33,35–37,41]. Baseline was the first sample collected either during acute infection (usually on the second day of hospitalization) for cholera patients, or before infection (Day 0) for experimentally infected volunteers [35]. The last follow-up day marked the end of study. Two studies found elevated vibriocidal antibody titers at 10 days and up to 9 months but returned to baseline by 1 year [24,33]. Titers returned to baseline earlier in 3 studies, by 3 [27,35] and 6 months [41] after acute infection. Four studies recorded significantly higher titers than at baseline for the entire 3 [36,37] and 6 [27,35] months of follow-up. Only one included persistence study stratified participants by age [27]. They found that elevated vibriocidal responses were shorter in children aged 2 to 5 (returning to baseline by 3 months) than in children aged 6 to 17 and adults.

Three older persistence studies analyzed the vibriocidal response of cholera patients in a descriptive manner [38–40]. In one study of 130 cholera patients in Bangladesh, vibriocidal antibody titers at least 4-fold higher were observed in 60% of patients 1 month after admission than at baseline and in 17% of patients at 6 months [38]. Similarly in a second study, mean titer levels were, by 12 to 18 months after hospitalization, only twice that observed at admission [39]. Elevated vibriocidal antibody titers were observed 9 to 11 months later in 11 individuals who had naturally acquired cholera (rather than experimentally) in the US [40]. Taken together, the duration of elevated vibriocidal antibody levels determined either statistically (Fig 3) or by titer fold differences [38–40] appear consistent, sharing a similar time course for patients in cholera endemic and nonendemic areas.

#### LPS- and OSP-specific IgG memory B cell responses

**Fig 4:**
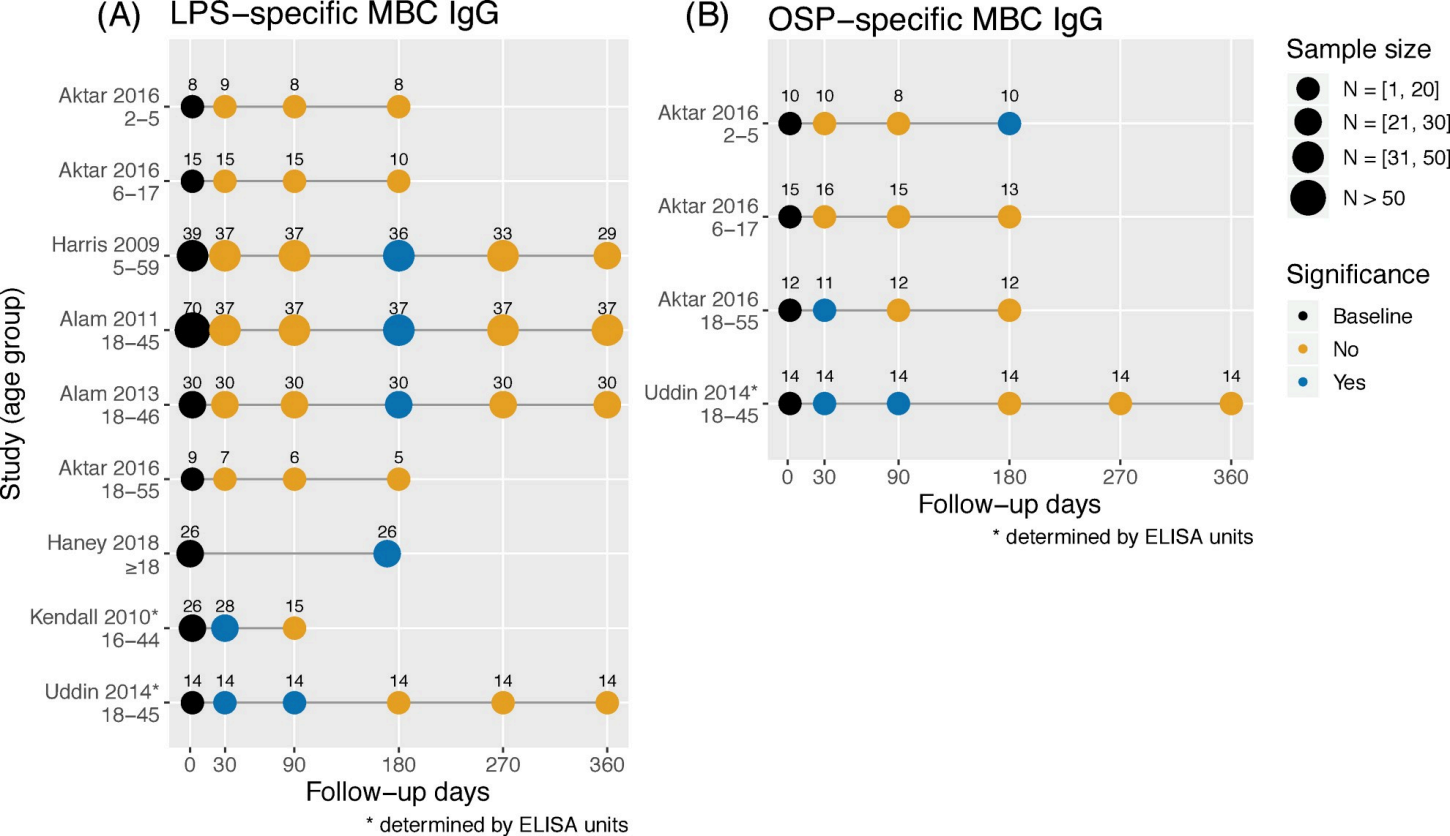
LPS- and OSP-specific IgG memory B cell responses by study and age range. Samples of LPS-specific IgG MBCs (A) and of OSP-specific IgG MBCs (B) were taken at different time points and compared for a statistically significant difference from baseline levels within a study group (*P* ≤ 0.05, two-tailed). A study is repeated in multiple lines if different age groups were considered. IgG, immunoglobulin G; LPS, lipopolysaccharide; MBC, memory B cell; OSP, O-specific polysaccharide.

Seven studies measured the LPS-specific IgG MBCs in circulation, using standardized enzyme-linked immunospot (ELISPOT) assays (quantified as a percentage of antigen-specific MBCs out of the total IgG MBCs) [21,24,27,33,34] or standardized enzyme-linked immunosorbent assay (ELISA) protocols [37,42] (Fig 4A). Strikingly, 3 studies measuring the MBC response of patients (children and adults) in Bangladesh with ELISPOT [24,33,34] with a 1-year follow-up showed an identical pattern: Samples were significantly higher than baseline at 6 months and returned to baseline by 9 months. In line with this pattern is the finding of samples of elevated LPS-specific IgG MBC levels from North American volunteers at 6 months [21]. This differs from one age-stratified study measuring MBC responses, which found no significant differences from baseline for all age groups throughout 6 months of follow-up.

Two included studies using ELISA protocols to measure the LPS-specific MBCs of IgG response offered a new picture, observing elevated levels from baseline at 1 month [37] or at 1 and 3 months [42] but returned to baseline afterwards (Fig 4A). This increase was not delayed as was observed in other studies [24,33,34].

Two included studies measured OSP-specific IgG MBC in circulation [27,42] (Fig 4B). Aktar and colleagues [27] found different OSP-specific IgG MBC response in children and adults. Particularly, children aged 2 to 5 showed a delayed increase at 6 months, while adults displayed an early increase at 1 month that returned to baseline by 3 months, and no difference to baseline were observed in children aged 6 to 17. Uddin and colleagues [42] measured the OSP-specific IgG MBC response in adults aged 18 to 45 and found the samples significantly different from baseline at 1 and 3 months but declined to baseline by 6 months. Taken together, these studies indicate that OSP-specific IgG MBC response stops being detectable only a few months following infection.

## Discussion

In this review, we synthesized multiple types of evidence to investigate the duration of protection following natural infection with *V*. *cholerae*. We identified consistencies and differences across studies of long-term epidemiological records, human challenge studies, and immune persistence studies that measured serological responses to *V*. *cholerae*.

Three out of 4 included observational studies found considerable protection following clinical cholera, with around 60% reduced risk of subsequent clinical cholera over 3 years. Glass and colleagues found repeat hospitalizations were 90% lower than expected [11]. Their finding contrasts with Woodward’s finding that an infection with *V*. *cholerae* (both symptomatic and asymptomatic) does not offer long-lasting immunity to repeat infections [43]. Woodward used data that included asymptomatic infections (detected by house-to-house visits), whereas Glass and colleagues used data from symptomatic patients with diarrhea. Given that as much as 75% of cholera infections may be asymptomatic [2,45,46], care must be taken to extrapolate the findings from symptomatic cholera infections to all cholera infections (including asymptomatic ones) as a whole.

Results from the observational studies aligned with those from the 3 challenge studies. One study found that clinical cholera conferred protection against subsequent cholera for at least 3 years, the longest interval tested. Two other studies supported this finding, observing that immunity had persisted throughout their entire 1-year follow-up. While these studies serve as evidence that symptomatic cholera protects against subsequent cholera, the evidence that asymptomatic infections of *V*. *cholerae* offer the same protection is lacking. It is plausible that asymptomatic infections offer a shorter and less robust protection than symptomatic ones. It is suggested that the dosage of antigen used in challenges may influence the infection experience and thus acquired immunity [47,48]. Yet, the dosage of *V*. *cholerae* used on participants varied across challenge studies. Challenge studies included the dosage of *V*. *cholerae* used for the second challenge but not the first. Additional details of the first challenge, such as the dosage used, would allow a more solid comparison and improved interpretation between the initial and subsequent challenges.

It is important to consider the cholera epidemiology, particularly the interplay between classical and El Tor biotypes, to add context to the results of the observational studies. In Bangladesh, the classical biotype was dominant up to 1972, disappeared between 1973 and 1978, and reemerged in 1982 to become the epidemic strain [49]. Since 1984, the classical biotype has been progressively displaced by the El Tor biotype as the epidemic strain [49]. By 1991, the classical biotype was rarely isolated, and El Tor became the predominant strain [49]. The study of Clemens and colleagues [12] took place when the classical biotype was disappearing, which brings into question whether the observed protection against subsequent disease from initial disease with classical *V*. *cholerae* was independent of the exit of classical cholera underway.

Our present study teased apart challenge studies of participants with and without symptoms upon initial challenge. This complements other reviews that included the acute immune response following natural infection or vaccination [50] or focused on the immune response in children [51].

Compared to observational and challenge studies, the immune persistence studies gave a different picture, showing that vibriocidal antibodies returned to baseline levels within 1 year. While baseline vibriocidal titers have been correlated with protection to infection of *V*. *cholerae* O1, infections still occurred in household contacts with high baseline vibriocidal antibody titers [22]. Despite being an incomplete marker, vibriocidal antibodies are still considered the best immune marker of protection against cholera [21,22,52].

LPS- and OSP-specific IgG MBCs have been suggested as possible markers of long-term immunity against cholera [26]. However, the studies identified here show that both markers become undetectable by 6 months after hospitalization or experimental infection. Hence, with the current available technology, these markers cannot be used as predictors of long-term immune protection. It has been hypothesized that serum and mucosal IgA—or perhaps multiple immune responses—may be important for protection [53,54], but it is unclear how these immune responses translate to protection.

In the present work, we concentrated on the duration of protection against cholera estimated directly from data. However, mathematical modeling studies have also estimated the duration of natural immunity for cholera, ranging weeks to as long as 9 years (with a strong degree of protection in the first 5 years and subsequent waning for the next four) [28–30]. The upper end of these estimates is consistent with Glass and colleagues [11], who found only 3 repeat hospitalizations over the course of 9 years. However, the longest demonstrated duration of immunity from a human challenge came from one study, in which 4 participants showed no symptoms in their third challenge with *V*. *cholerae*, at 3 years after their initial challenge [17]. The lower end of these estimates is consistent with another challenge study, in which the majority of participants experienced diarrhea upon their second challenge only 4 to 12 months after the initial challenge [14]. Mathematical models are increasingly used to evaluate and design public health policy [55–58]. Because model parameters may greatly shape model outcomes, accurate parameter estimates are crucial. In particular, an imprecise estimate of protection for cholera could lead to a suboptimal use of valuable resources, such as vaccines.

Indeed, in recent years, oral cholera vaccines have become an additional tool to fight explosive cholera outbreaks around the world [1]. Several studies suggest that vaccine-induced immunity lasts for at least 3 years [59,60]. Hence, it is expected that natural immunity would be at least equally long, pointing to a longer infection-acquired protection from cholera than detected in serological studies. Understanding the duration of both natural and vaccine-induced immunity will be important as vaccines are administered over an outbreak in an endemic setting.

We identified 3 challenge studies in which most participants without diarrhea on initial challenge developed symptoms upon rechallenge. While comprising only 8 participants, these challenge studies present some evidence that subclinical infections may confer weaker long-term protection than clinical infections. There is evidence that seroconversion occurred less often in participants with subclinical infections than in those with clinical ones [61,62]. Further studies are required to elucidate whether clinical cholera induces a more robust and durable immune response than a subclinical infection given that as much as 75% of cholera infections are asymptomatic [2,45,46].

We make 3 notable observations here. First, doses of *V*. *cholerae* and definitions of diarrhea varied across challenge studies, adding variability. Additionally, whereas dosage and buffering are controlled in challenge studies, neither the dosage nor the stomach acidity are known in cholera patients. Other confounding variables influencing the risk and disease severity of a subsequent infection include malnutrition, genetics, and hypochlorhydria [20,63]. Second, each country with endemic cholera has its own set of transmission dynamics resulting in different levels of natural boosting. Yet, the majority of observational and persistence studies in this review were done with hospitalized patients in Bangladesh, where frequent exposure to *V*. *cholerae* may boost the immune response and thus prolong protection. Studies of household contacts of cholera patients in Bangladesh found potential immunological markers associated with decreased risk of infection [20,32]. Indeed, uninfected household contacts of cholera patients from Bangladesh were found to have higher baseline levels of certain immunological markers than infected contacts [32]. Volunteers in challenge studies from North America, however, likely had no natural boosting. More studies in different settings can help to translate these results to other endemic and nonendemic areas. Third, all challenge studies except one were done during the classical biotype era. The classical biotype has since been displaced by the El Tor biotype. The features of immunity following infection shared across biotypes are unknown.

Because protection against *V*. *cholerae* lasts longer than detectable elevated levels in serum antibodies, it has been hypothesized that protective immunity may be mediated by anamnestic responses of MBCs in the gut-associated lymphoid tissue or in the blood [24,64]. Indeed, mucosal immune responses have been observed in cholera patients, as measured by elevated levels of antibody-secreting cells (ASCs) and lymphocyte supernatants. Comparison of ASC levels and lymphocyte supernatants in circulation and in mucosal tissues over time may improve understanding of the potential roles of systemic and mucosal responses to provide protective immunity against *V*. *cholerae*.

This review of potential immunological markers, challenge, and observational studies of cholera has revealed the uncertainties and evidence gaps in our understanding of duration of infection-acquired immunity for cholera. We have presented a discrepancy between the estimated duration of immunity from cholera infection and the supporting serological evidence. Further, we have presented evidence that subclinical infections might result in less robust protection than clinical ones. More studies with follow-ups to asymptomatic participants are needed to bridge these gaps. A clear understanding of the duration of infection-acquired cholera immunity will help shape strategies for cholera control.

**Key Learning Points**

- Cholera is a diarrheal infectious disease that is associated with a high global burden.
- The estimated duration of protection from cholera ranges widely from months to 9 years.
- The duration and degree of protection induced by subclinical infections of *V*. *cholerae* may be shorter and less robust than those induced by clinical ones.
- Developing a better understanding the duration and degree of protection acquired from clinical and subclinical infections of *V*. *cholerae* is needed to guide control strategies and policy.

**Top five papers**

1. Levine MM, Black RE, Clements ML, Cisneros L, Nalin DR, Young CR. Duration of infection-derived immunity to cholera. J Infect Dis. 1981;143(6):818–20.
2. Ali M, Emch M, Park JK, Yunus M, Clemens J. Natural cholera infection-derived immunity in an endemic setting. J Infect Dis. 2011;204(6):912–8.
3. Cash RA, Music SI, Libonati JP, Craig JP, Pierce NF, Hornick RB. Response of man to infection with *Vibrio cholerae*. II. Protection from illness afforded by previous disease and vaccine. J Infect Dis. 1974;130(4):325–33.
4. Clemens JD, van Loon F, Sack DA, Rao MR, Ahmed F, Chakraborty J, et al. Biotype as determinant of natural immunising effect of cholera. Lancet. 1991;337(8746):883–4.
5. Glass RI, Becker S, Huq MI, Stoll BJ, Khan MU, Merson MH, et al. Endemic cholera in rural Bangladesh, 1966–1980. Am J Epidemiol. 1982;116(6):959–70.

## Supporting information

S1 Appendix Studies of other immunological markers Fig A. **Studies measuring LPS-specific IgA and IgM memory B cells by study and age range.** Samples of LPS-specific IgA (A) and IgM (B) memory B cells were taken at different time points and compared for a statistically significant difference from baseline levels within a study group (*P* ≤ 0.05, two-tailed). **Fig B. Studies measuring OSP-specific IgA and IgM memory B cells by study and age range.** Samples of OSP-specific IgA (A) and IgM (B) memory B cells were taken at different time points and compared for a statistically significant difference from baseline levels within a study group (*P* ≤ 0.05, two-tailed). **Fig C. Studies measuring CTB-specific IgA, IgG, and IgM memory B cells by study and age range.** Samples of CTB-specific IgA (A), IgG (B), and IgM (C) memory B cells were taken at different time points and compared for a statistically significant difference from baseline levels within a study group (*P* ≤ 0.05, two-tailed). CTB, B subunit of cholera toxin; LPS, lipopolysaccharide; OSP, O-specific polysaccharide.
(DOCX)
