# Supplementary material for: Protection afforded by previous Vibrio cholerae infection against subsequent disease and infection: A review
Source: PLoS Negl Trop Dis. 2021 May 20;15(5):e0009383. doi: 10.1371/journal.pntd.0009383 (PMC8136710; doi:10.1371/journal.pntd.0009383)
Supplement: S1 Appendix — Studies measuring LPS-specific IgA and IgM memory B cells by study and age range. Samples of LPS-specific IgA (A) and IgM (B) memory B cells were taken at different time points and compared for a statistically significant difference from baseline levels within a study group (P ≤ 0.05, two-tailed). Fig B. Studies measuring OSP-specific IgA and IgM memory B cells by study and age range. Samples of OSP-specific IgA (A) and IgM (B) memory B cells were taken at different time points and compared for a statistically significant difference from baseline levels within a study group (P ≤ 0.05, two-tailed). Fig C. Studies measuring CTB-specific IgA, IgG, and IgM memory B cells by study and age range. Samples of CTB-specific IgA (A), IgG (B), and IgM (C) memory B cells were taken at different time points and compared for a statistically significant difference from baseline levels within a study group (P ≤ 0.05, two-tailed). CTB, B subunit of cholera toxin; LPS, lipopolysaccharide; OSP, O-specific polysaccharide. (DOCX) [file pntd.0009383.s001.docx]

**Supporting Information**

**Title:** Protection afforded by previous *Vibrio cholerae* infection against subsequent disease and infection: a review

**Authors:** Tiffany Leung^a^, Laura Matrajt^a*^

**^a^** Vaccine and Infectious Diseases Division, Fred Hutchinson Cancer Research Center, Seattle, Washington, United States of America

^*^ Corresponding author: laurama@fredhutch.org

**S1 Appendix**

**Studies of other immunological markers**

Responses of LPS-specific IgA and IgM memory B cells were recorded in seven included studies (Fig A). Three of four studies with a one-year follow-up showed measurements of LPS-specific IgA MBCs with a similar pattern: they peaked at one month, returned to baseline at three and six months, elevated again at nine months before returning to baseline at one year (Fig A) [1–3]. The responses of LPS-specific IgM MBCs recorded in two studies peaked at one month [4] or one and three months [5] before returning to baseline (Fig A).

The OSP-specific IgA and IgM memory-B cell responses were recorded in two studies from our search. Levels of OSP-specific IgA MBC generally peaked at one or three months and returned to baseline by six months (Fig B). Patterns of OSP-specific IgM MBCs were different: One [5] found elevated levels at one and three months that returned to baseline by six months. The other study [6] found no significant difference from baseline over six months of follow-up for young children and adults. However, for children between 6 and 17 years old, OSP-specific IgM MBC levels were significantly higher at three months and returned to baseline by six months (Fig B).

The responses of CTB-specific IgG MBCs lasted the longest (significantly higher than baseline at all time points over one year, in three studies) compared to responses of CTB-specific IgA and IgM memory B cell response (Fig C). However, in studies of household contacts of patients with cholera in Bangladesh, no long-term protection has been associated with CTB-specific IgA and IgG MBCs or LPS-specific IgA MBCs [7].

**References**

1. Harris AM, Bhuiyan MS, Chowdhury F, Khan AI, Hossain A, Kendall EA, et al. Antigen-specific memory B-cell responses to Vibrio cholerae O1 infection in Bangladesh. Infect Immun. 2009;77(9):3850–6.

2. Alam MM, Riyadh MA, Fatema K, Rahman MA, Akhtar N, Ahmed T, et al. Antigen-specific memory B-cell responses in Bangladeshi adults after one- or two-dose oral killed cholera vaccination and comparison with responses in patients with naturally acquired cholera. Clin Vaccine Immunol. 2011;18(5):844–50.

3. Alam MM, Arifuzzaman M, Ahmad SM, Hosen MI, Rahman MA, Rashu R, et al. Study of avidity of antigen-specific antibody as a means of understanding development of long-term immunological memory after Vibrio cholerae O1 infection. Clin Vaccine Immunol. 2013;20(1):17–23.

4. Kendall EA, Tarique AA, Hossain A, Alam MM, Arifuzzaman M, Akhtar N, et al. Development of immunoglobulin M memory to both a T-cell-independent and a T-cell-dependent antigen following infection with Vibrio cholerae O1 in Bangladesh. Infect Immun. 2010;78(1):253–9.

5. Uddin T, Aktar A, Xu P, Johnson RA, Rahman MA, Leung DT, et al. Immune responses to O-specific polysaccharide and lipopolysaccharide of Vibrio cholerae O1 Ogawa in adult Bangladeshi recipients of an oral killed cholera vaccine and comparison to responses in patients with cholera. Am J Trop Med Hyg. 2014;90(5):873–81.

6. Aktar A, Rahman MA, Afrin S, Faruk MO, Uddin T, Akter A, et al. O-specific polysaccharide-specific memory B cell responses in young children, older children, and adults infected with Vibrio cholerae O1 Ogawa in Bangladesh. Clin Vaccine Immunol. 2016;23(5):427–35.

7. Patel SM, Rahman MA, Mohasin M, Riyadh MA, Leung DT, Alam MM, et al. Memory B cell responses to Vibrio cholerae O1 lipopolysaccharide are associated with protection against infection from household contacts of patients with cholera in Bangladesh. Clin Vaccine Immunol. 2012;19(6):842–8.

**Fig A. Studies measuring LPS-specific IgA and IgM memory B cells by study and age range.** Samples of LPS-specific IgA (A) and IgM (B) memory B cells were taken at different time points and compared for a statistically significant difference from baseline levels within a study group (*P* ≤ 0.05, two-tailed).

**Fig B. Studies measuring OSP-specific IgA and IgM memory B cells by study and age range.** Samples of OSP-specific IgA (A) and IgM (B) memory B cells were taken at different time points and compared for a statistically significant difference from baseline levels within a study group (*P* ≤ 0.05, two-tailed).

**Fig C. Studies measuring CTB-specific IgA, IgG, and IgM memory B cells by study and age range.** Samples of CTB-specific IgA (A), IgG (B), and IgM (C) memory B cells were taken at different time points and compared for a statistically significant difference from baseline levels within a study group (*P* ≤ 0.05, two-tailed).
